# Effects of preoperative high-oral protein loading on short- and long-term renal outcomes following cardiac surgery: a cohort study

**DOI:** 10.1186/s12967-022-03410-x

**Published:** 2022-05-10

**Authors:** Faeq Husain-Syed, David R. Emlet, Jochen Wilhelm, Tommaso Hinna Danesi, Fiorenza Ferrari, Pércia Bezerra, Salvador Lopez-Giacoman, Gianluca Villa, Khodr Tello, Horst-Walter Birk, Werner Seeger, Davide Giavarina, Loris Salvador, Dana Y. Fuhrman, John A. Kellum, Claudio Ronco, the IRRIV-AKI Study Group, Carlotta Caprara, Valentina Corradi, Massimo Cal, Carla Estremadoyro, Renhua Lu, Sara Samoni, Aashish Sharma, Lorenzo Tofani, Grazia Maria Virzì

**Affiliations:** 1 Department of Nephrology, Dialysis and Transplantation, International Renal Research Institute of Vicenza, San Bortolo Hospital, Via Rodolfi, 37, 36100 Vicenza, Italy; 2 Department of Internal Medicine II, University Hospital Giessen and Marburg, Justus-Liebig-University Giessen, Klinikstrasse 33, 35392 Giessen, Germany; 3 Center for Critical Care Nephrology, CRISMA, Department of Critical Care Medicine, School of Medicine, University of Pittsburgh, 3550 Terrace Street, Pittsburgh, PA 15261 USA; 4 Institute for Lung Health, Justus-Liebig-University Giessen, Ludwigstrasse 23, 35390 Giessen, Germany; 5 Department of Cardiac Surgery, San Bortolo Hospital, Via Rodolfi, 37, 36100 Vicenza, Italy; 6 Intensive Care Unit, I.R.C.C.S. Policlinico San Matteo, Viale Camillo Golgi, 19, 27100 Pavia, Italy; 7 Division of Cardiac Surgery, Department of Surgery, College of Medicine, University of Cincinnaci, 231 Albert Sabin Way, Cincinnati, OH 45267-0558 USA; 8 Department of Health Science, Section of Anesthesiology and Intensive Care, University of Florence, Piazza San Marco, 4, 50121 Florence, Italy; 9 Member of the German Centre for Lung Research, Universities of Giessen and Marburg Lung Centre, Klinikstrasse 33, 35392 Giessen, Germany; 10 Department of Lung Development and Remodelling, Max Planck Institute for Heart and Lung Research, Ludwigstrasse 43, 61231 Bad Nauheim, Germany; 11 Department of Clinical Chemistry and Hematology Laboratory, San Bortolo Hospital, Via Rodolfi, 37, 36100 Vicenza, Italy; 12 Departments of Critical Care Medicine and Pediatrics, Children’s Hospital of University of Pittsburgh Medical Center, One Children’s Hospital Way, 4401 Penn Ave, Pittsburgh, PA 15224 USA; 13 Department of Medicine (DIMED), Università di Padova, Via Giustiniani, 2, 35128 Padua, Italy; 14 Department of Neurosciences, Psychology, Drug Research and Child Health, University of Florence, Piazza San Marco, 4, 50121 Florence, Italy

**Keywords:** Acute kidney injury, Chronic kidney disease, Kidney stress test, Renal recovery

## Abstract

**Background:**

Post-cardiac surgery acute kidney injury (AKI) is associated with increased mortality. A high-protein meal enhances the renal blood flow and glomerular filtration rate (GFR) and might protect the kidneys from acute ischemic insults. Hence, we assessed the effect of a preoperative high-oral protein load on post-cardiac surgery renal function and used experimental models to elucidate mechanisms by which protein might stimulate kidney-protective effects.

**Methods:**

The prospective “Preoperative Renal Functional Reserve Predicts Risk of AKI after Cardiac Operation” study follow-up was extended to postoperative 12 months for 109 patients. A 1:2 ratio propensity score matching method was used to identify a control group (n = 214) to comparatively evaluate the effects of a preoperative protein load and standard care. The primary endpoints were AKI development and postoperative estimated GFR (eGFR) loss at 3 and 12 months. We also assessed the secretion of tissue inhibitor of metalloproteases-2 (TIMP-2) and insulin-like growth factor–binding protein 7 (IGFBP7), biomarkers implicated in mediating kidney-protective mechanisms in human kidney tubular cells that we exposed to varying protein concentrations.

**Results:**

The AKI rate did not differ between the protein loading and control groups (13.6 vs. 12.3%; p = 0.5). However, the mean eGFR loss was lower in the former after 3 months (0.1 [95% CI − 1.4, − 1.7] vs. − 3.3 [95% CI − 4.4, − 2.2] ml/min/1.73 m^2^) and 12 months (− 2.7 [95% CI − 4.2, − 1.2] vs − 10.2 [95% CI − 11.3, − 9.1] ml/min/1.73 m^2^; p < 0.001 for both). On stratification based on AKI development, the eGFR loss after 12 months was also found to be lower in the former (− 8.0 [95% CI − 14.1, − 1.9] vs. − 18.6 [95% CI − 23.3, − 14.0] ml/min/1.73 m^2^; p = 0.008). A dose–response analysis of the protein treatment of the primary human proximal and distal tubule epithelial cells in culture showed significantly increased IGFBP7 and TIMP-2 expression.

**Conclusions:**

A preoperative high-oral protein load did not reduce AKI development but was associated with greater renal function preservation in patients with and without AKI at 12 months post-cardiac surgery. The potential mechanisms of action by which protein loading may induce a kidney-protective response might include cell cycle inhibition of renal tubular epithelial cells.

*Clinical trial registration* ClinicalTrials.gov: NCT03102541 (retrospectively registered on April 5, 2017) and ClinicalTrials.gov: NCT03092947 (retrospectively registered on March 28, 2017).

**Supplementary Information:**

The online version contains supplementary material available at 10.1186/s12967-022-03410-x.

## Background

Acute kidney injury (AKI) is the most common major complication of cardiac surgery and independently associated with increased short- and long-term morbidity and mortality [1, 2]. Although many patients show AKI reversal within days to weeks of development, data suggest a strong association between AKI and subsequent chronic kidney disease (CKD) [3, 4]. Hence, effective approaches to prevent the development of AKI are vital for reducing the burden of CKD. The pathophysiology of AKI after cardiac surgery is likely characterized by the involvement of multiple injury pathways to varying degrees in patients preoperatively, intraoperatively, and postoperatively [5]. Among the proposed mechanisms, alterations in renal perfusion during cardiopulmonary bypass and postoperative renal ischemic injury are thought to be important contributors to AKI [6].

In healthy humans and adults scheduled to undergo cardiac surgery, a high protein load reportedly enhances the glomerular filtration rate (GFR) through a global increase in renal blood flow due to afferent arteriolar dilation and a decrease in renal vascular resistance resulting in glomerular hyperfiltration [7]. This presumably happens via the modulation of the tubuloglomerular feedback mechanism and changes in the levels of vasoactive compounds [8, 9]. In contrast, protein restriction is associated with a reduction in renal blood flow and GFR [10]. Animal models has shown that amino acids can protect the kidneys from acute ischemic insults, likely by maintaining the renal blood flow and thus ameliorating ischemic and inflammatory injuries that could contribute to AKI [11].

However, interventions that increase the renal blood flow (e.g., dopamine application) have been uniformly ineffective in preventing or treating AKI. A possible reason is that the protein load may stress the kidneys in the same way that direct or remote ischemic preconditioning (RIPC) may [12, 13]. This sub-injurious “stress” may trigger protective mechanisms in the kidneys. The release of tissue inhibitor of metalloproteases-2 (TIMP-2) and insulin-like growth factor–binding protein 7 (IGFBP7), which induce cell cycle arrest, appears to be a marker of this protective response [12, 13].

We previously reported the value of the renal functional reserve (RFR), measured based on a preoperative high-oral protein load, for the prediction of AKI in patients undergoing elective cardiac surgery [14]. In the present study, we sought to examine whether protein loading *itself* may affect the development of AKI and long-term renal function after cardiac surgery in comparison with that in matched controls with similar demographic, risk, and surgical profiles who received standard preoperative care. Furthermore, we used in vitro cell culture models involving primary human kidney proximal and distal tubule cells to elucidate the potential mechanism by which protein might stimulate kidney-protective effects directly at the tubular level.

## Methods

### Study design and participants

This single-center, non-blinded, cohort study included patients enrolled in the prospective “Preoperative RFR Predicts Risk of AKI after Cardiac Operation” study [14]. The study included 110 adults with eGFR [15] values ≥ 60 ml/min/1.73 m^2^ scheduled to undergo elective cardiac surgery (coronary artery bypass, valve replacement, a combination of the two, or other surgery, with cardiopulmonary bypass) between November 2014 and October 2015 at the Department of Cardiac Surgery, San Bortolo Hospital, Vicenza, Italy. Detailed methods including surgical care are described in the Additional file 1. Notably, patients who did not discontinue treatment with renin–angiotensin-system blockers (part of the standard procedure at the Department of Cardiac Surgery in Vicenza), within a minimum of 48 h before admission were excluded.

The study protocol has been described in detail elsewhere [14]. Briefly, patients received a high-oral protein load (1.2 g/kg bodyweight of unsalted red cooked meat protein without additives) to assess their RFR the day before surgery in addition to standard preoperative care [14] and at 3 months post-surgery in the outpatient setting [16]. Approximately one week prior to RFR assessment, participants were given exact instructions about medication (no nonsteroidal anti-inflammatory drugs, no renin–angiotensin-system blockers) that were allowed on the morning of the examination. Estimates of the potential effect and variance of protein intake on GFR were obtained from previous publications [17, 18]. Furthermore, the high-oral protein load was defined according to a recent dose testing, which suggested a ceiling effect with an upper limit of maximal obtainable GFR following a high-oral protein intake ≥ 1 g/kg bodyweight [19]. Results of the bioimpedance vector analysis in the protein loading group are provided in the Additional file 1: Table S1. The control group was identified through a propensity score analysis conducted on the covariates of age, gender, race, cardiac disease, and baseline eGFR undergoing similar procedures around the period during which the RFR cohort underwent surgery (range: 0–3 months) using a 1:2 ratio matching method (for other eligibility criteria see Additional file 1). The control patients received standard preoperative care, which was defined pragmatically. The study was approved by the local ethics committee (79/16F) and complied with the tenets of the Declaration of Helsinki. The patients were approached for enrollment during admission consultations and provided written informed consent.

## Procedures and measurements

### Evaluation of kidney function

AKI was defined according to the KDIGO workgroup criteria as an increase in SCr by ≥ 0.3 mg/dl within 48 h or ≥ 1.5-times the baseline value within 7 days or urine output < 0.5 ml/kg/h for 6 h [20]. Baseline SCr was determined based on all available SCr values from hospital and outpatient medical records within the previous 90 days. Those whose baseline SCr level data were unavailable, we expanded the screening criteria to an increase or decrease in SCr by 0.3 mg/dL during the hospital stay. Positive fluid balance and hemodilution are considered in the diagnosis and staging of AKI using the following formula [21]:

$${\text{Adjusted SCr level}}\, = \,{\text{SCr}}\, \times \,{\text{correction factor}}$$

where the correction factor = (weight (kg) upon hospital admission × 0.6 + Σ (daily cumulative fluid balance (liter)))/hospital admission weight × 0.6

AKI reversal was defined as the absence of any stage of AKI at hospital discharge [22]. All cases were reviewed on a case-by-case basis to confirm each diagnosis of AKI and AKI reversal by a blinded adjudication committee comprising three expert nephrologists blinded to clinical data. If two reviewers disagreed, a third reviewer provided input and a consensus was developed. Spot urine for albuminuria were collected prior to surgery, and at 3 and 12 months of follow-up. Albuminuria was normalized to the urinary creatinine concentration to account for urine dilution. Information on sample collection and laboratory methods are provided in Additional file 1.

### Follow-up and endpoints

Clinical outcomes were evaluated for 1 year after discharge. Changes in medication for clinical reasons were permitted. At 3 and 12 months of follow-up, analyses were conducted at the Outpatient Nephrology Department, and patients were contacted via the telephone 1 week before the follow-up analyses and advised to maintain their usual dietary habits and avoid high dietary protein intake (> 0.8 g/kg body weight per day). A high-oral protein diet during the follow-period was not intended as per study protocol. Patients were again advised to suspend renin–angiotensin-system blockers and non-steroidal inflammatory drugs a minimum of 48 h prior to outpatient presentation. The primary endpoint was AKI within 7 days post-cardiac surgery. Additional measures included the difference in eGFR at 3 and 12 months post-surgery compared to the preoperative values (Additional file 1: Fig. S2). The use of any potentially nephrotoxic drugs was recorded during the hospital stay.

### Protein load and biomarker secretion in human kidney tubule cells

Secretion of the AKI biomarkers IGFBP7 and TIMP-2 after protein loading was assessed using our established in vitro model systems of primary human proximal and distal tubule cells [23]. Immunoaffinity isolated proximal and distal tubule cells were cultured at confluence on Transwell permeable supports in hormonally defined, serum-free media to form epithelial monolayers. The cells were then treated on the apical side with increasing concentrations (0, 2.5, 5, 10, 20, and 50 µM) of protein (Life Extension, Wellness Code® Whey Protein Concentrate; amino acid profile provided in Additional file 1) for 6 h at 37 °C in 5% CO_2_. Apical conditioned media were subjected to SDS-PAGE and immunoblot analysis to detect secreted IGFBP7 and TIMP-2 by using goat polyclonal antibodies provided by Astute Medical Inc. Raw immunoblot signals were normalized to the lysate concentration, and the signal from whey-treated cells was compared to that from untreated cells and graphs were generated. Four separate donor isolates to assess IGFBP7 and three separate isolates to assess TIMP-2 were used. The average µM concentration of whey protein was based upon the molecular weight of β-lactoglobulin.

### Statistical analysis

Descriptive variables were expressed as median (interquartile range) or means [95% confidence interval] for continuous variables, and n (%) for categorical variables. Differences in the distributions between groups were tested using Mann–Whitney *U* tests for numeric variables and chi-squared tests for categorical variables. Changes in eGFR and SCr were calculated in patients as the difference in the values at follow-up and 1 day before surgery (before protein loading). Analyses were adjusted for age, sex, body mass index, hypertension, and diabetes. Conditional distributions of the response variables showed a sufficiently good approximation to the normal distribution, as assessed using normal quantile–quantile plots. Data were analyzed using R 3.6.0 (R Center for Statistical Computing, Vienna, Austria) [24]. Two-tailed p values < 0.05 were considered to indicate statistical significance. Sample size estimation was based on a recent observational study in a similar patient population [25] and on the primary endpoint (i.e., AKI development within 7 days post-cardiac surgery). Using the Fisher’s exact test, we calculated that 339 patients (226 patients in the control group and 113 patients in the protein-loading group) would be required to provide 80% power at a type 1 error of 5% to detect an AKI poportion of 0.2 and 0.05 and a group weight of 2:1, respectively. Nearest neighbor matching with a propensity score caliper distance of 0.1 was employed to select patients treated with standard care to be included in the control group. A 1:2 ratio matching method was used. An optimal quality match was defined as a standardized mean difference (SMD) ≤ 0.1 per matching variable between patients in the study and control groups. The time course of eGFR values relative to preoperative values was analyzed with a linear mixed model by using the logarithm of the relative eGFR values. The differences for categorical variables between groups were analyzed using Fisher’s exact test. For the in vitro studies, immunoblot films were imaged and quantitated using ImageJ and the paired *t*-test function of GraphPad Prism 8.0 (GraphPad Software, La Jolla, CA).

## Results

### Baseline characteristics

Of the 540 patients screened for the study, 324 were enrolled and included in the analysis. 110 patients received preoperative high-oral protein loading as part of the RFR study, whereas 214 patients were matched to the protein-loading group (Additional file 1: Fig. S1). The matching quality was considered excellent, as reflected by an SMD ≤ 0.1 for all matching variables. The baseline characteristics and surgical data were similar between both groups (Table 1). The eGFR values at hospital admission were similar in both the protein loading and control groups (90 [95% confidence interval (CI) 87–92] vs. 90 ml/min/1.73 m^2^ [95% CI 88–92], respectively; p = 0.914).

**Table 1:** Demographic and clinical characteristics of the cohort

	Control group^†^ (n = 214)	Protein-loading group^†^ (n = 110)	p-value
Demographics			
Age, years	63 (54 – 71)	62 (54 – 71)	0.693
Male sex, n (%)	151 (71%)	78 (71%)	0.948
Race/ethnicity, n (%)			0.856
White	210 (98.1%)	108 (98.2%)	
Black	4 (1.9%)	3 (1.8%)	
Weight, kg	76 (65 – 85)	77 (68 – 84)	0.431
Body mass index, kg/m^2^	24.6 (22.2 – 28.3)	25.5 (23.5 – 28.2)	0.134
Comorbidities, n (%)			
Hypertension	142 (66.4%)	72 (65.5%)	0.871
Atrial fibrillation	29 (13.6%)	24 (21.8%)	0.057
Peripheral vasculopathy	19 (8.5%)	8 (7.3%)	0.620
Type 2 diabetes mellitus	15 (7.0%)	7 (6.4%)	0.827
Smoking status (former smoker or current smoker)	76 (35.5%)	40 (36.4%)	0.745
Dyslipidemia	65 (30.4%)	25 (22.7%)	0.146
Medication, n (%)			
Antiplatelet	38 (17.8%)	18 (16.4%)	0.753
Beta-blocker	108 (50.5%)	41 (37.3%)	0.024
ACEi or ARB	99 (46.3%)	50 (45.5%)	0.890
Statin	50 (23.4%)	39 (35.5%)	0.021
Diuretic^a^	57 (26.6%)	27 (24.5%)	0.684
Baseline clinical data			
Leucocytes, × 10^9^/L	6.40 (5.30 – 7.40)	6.35 (5.43 – 7.62)	0.727
Hemoglobin, g/dL	14.2 (13.2 – 15.2)	14.0 (13.1 – 14.8)	0.198
Platelets, × 10^9^/L	204 (176 – 241)	212 (184 – 246)	0.186
Albumin, g/dL	4.0 (3.8 – 4.1)	3.9 (3.8 – 4.1)	0.445
eGFR, mL/min/1.73 m^2 b^	90 (80 − 98)	91 (82 − 100)	0.886
Urea, mg/dL^c^	35.0 (30.0 – 41.3)	36.0 (29.0 – 43.0)	0.392
Troponin I, μg/L	0.01 (0.01 – 0.01)	0.01 (0.01 – 0.01)	0.328
NYHA classification, n (%)			0.697
1	75 (35.4%)	43 (39.1%)	
2	133 (62.1%)	65 (59.1%)	
3	6 (2.8%)	2 (1.8%)	
Left ventricular ejection fraction, %	62.9 (58.0 – 68.0)	61.0 (58.0 – 66.0)	0.449
Systolic pulmonary arterial pressure, mm Hg	30.0 (28.0 − 37.0)	30.0 (22.3 − 38.0)	0.334
EuroSCORE II for operative risk, %^d^	1.17 (0.85 − 1.99)	1.03 (0.69 − 1.83)	0.294
STS risk score, %^e^			
Risk of mortality	0.64 (0.34 – 1.13)	0.60 (0.41 – 1.36)	0.423
Risk of morbidity or mortality	8.55 (6.50 − 10.87)	8.47 (6.66 − 12.25)	0.389
Risk of renal failure	1.34 (0.90 − 2.22)	1.29 (0.90 − 2.07)	0.847
Thakar score^f^			0.326
0.4	165 (77.1%)	90 (81.8%)	
1.8	49 (22.9%)	20 (18.2%)	
Operative data			
Aortic cross-clamp, min	79.5 (59.0 − 97.0)	79.5 (60.0 − 104.5)	0.445
Cardiopulmonary bypass time, min	112.5 (90.0 − 138.0)	115.0 (98.0 − 149.8)	0.182
Procedure, n (%)			
Coronary artery bypass graft only	8 (3.8%)	4 (3.6%)	0.507
Valve only	107 (50.5%)	58 (52.7%)	0.457
Combined or other	97 (45.8%)	48 (43.6%)	0.142
Minimally invasive	141 (66%)	72 (65%)	0.468
Intraoperative diuresis, mL	1000 (550 − 1377)	800 (552 − 1200)	0.252
Surgery fluid balance, mL	3650 (2880 − 4327)	3815 (2892 − 4537)	0.378
Lowest mean arterial pressure, mmHg	66.7 (63.3 − 66.7)	66.7 (63.3 − 73.3)	0.007
Lowest hemoglobin, g/dL	10.5 (8.9 − 10.9)	9.6 (8.7 − 10.8)	0.329
Red blood cell transfusion, n (%)	6 (2.8%)	11 (10.0%)a	0.012
ICU data			
Mechanical ventilation, days	1.0 (1.0 − 1.0)	1.0 (1.0 − 1.0)	0.445
Intra-aortic balloon pump, n (%)	5 (2.3%)	3 (2.7%)	0.830
Extra-corporeal membrane oxygenation, n (%)	2 (0.9%)	1 (0.9%)	0.982
Myocardial infarction, n (%)	0 (0%)	1 (0.9%)	0.501
Stroke, n (%)	1 (0.5%)	3 (2.7%)	0.081
Re-intervention, n (%)	6 (2.8%)	2 (1.8%)	0.588
Day 1 fluid balance, mL	− 718 (− 1274 to − 117)	− 150 (− 640 to 301)	0.305
Day 2 fluid balance, mL	− 150 (− 640 to 301)	− 270 (− 1020 to 115)	0.022
Weight difference, kg^g^	− 1.50 (− 3.00 to − 0.10)	- 2.05 (− 3.40 to − 0.02)	0.170
ACEi or ARB use, n (%)	53 (24.8%)	22 (20.0%)	0.335
Aminoglycoside use, n (%)	2 (0.9%)	0 (0%)	0.309
Vancomycin use, n (%)	3 (1.4%)	0 (0%)	0.212
NSAID drug use, n (%)	4 (1.9%)	0 (0%)	0.149
Mean arterial pressure < 65 mmHg within the first 24 h, n (%)	83 (38.8%)	35 (31.8%)	0.217
Inotropes, n (%)	67 (31.3%)	41 (37.3%)	0.281
ICU stay, h	57 (41 − 87)	51 (41 − 71)	0.569
Hospital stay, days	6 (6 − 8)	5 (4 − 6)	< 0.001
3-month follow-up data			
ACEi or ARB	125 (58.4%)	65 (59.6%)	0.435
Readmission, n (%)	4 (1.9%)	5 (4.5%)	0.165
3-month mortality, n (%)	0 (0%)	1 (0.9%)	0.162
1-year follow-up data			
ACEi or ARB	112 (52.8%)	59 (54.1%)	0.392
1-year mortality, n (%)	2 (0.9%)	1 (0.9%)	0.982

Covariates used in the propensity score analysis (age, gender, race, cardiac disease, baseline eGFR) did not show significant differences between the protein loading and control groups

*ACEi* angiotensin-converting enzyme inhibitor, *ARB* angiotensin II receptor blocker, *ICU* intensive care unit, *IQR* interquartile range, *NSAID* non-steroidal anti-inflammatory drug; *NYHA* New York Heart Association, *STS* Society of Thoracic Surgeons

^†^Summaries of quantitative variables are presented as median and interquartile range (in parentheses). For categorical variables, the absolute and relative frequencies (as %, in parentheses) for the categories are presented

^a^Diuretics include loop diuretics and thiazides

^b^The eGFR was calculated using the Chronic Kidney Disease Epidemiology Collaboration equation [15]

^c^To convert the value for urea to blood urea nitrogen, it was multiplied by 0.467

^d^The European System for Cardiac Operative Risk Evaluation (EuroSCORE) score is calculated using a logistic-regression equation and ranges from 0 to 100%, with higher scores indicating greater risk

^e^The STS risk score is calculated using a logistic-regression equation; it estimates the risk of morbidity and mortality and the risk of renal failure, and ranges from 0 to 100% (higher scores indicate greater risk)

^f^The Thakar score is calculated using a logistic-regression equation; it estimates the risk of dialysis for patients undergoing cardiac surgery, and ranges from 0% to 21.5% (higher scores indicate greater risk)

^g^ICU discharge − hospital admission

### AKI rates

The rate of AKI was not different between the protein loading and control groups (15/110 [13.6%] vs 26/214 [12.1%], p = 0.5; using full AKI staging: p = 0.945; Table 2). All patients who developed AKI experienced only one distinct episode of AKI throughout the hospital stay. The correction of SCr for fluid balance did not affect the diagnosis and staging of AKI. All 15 patients in the protein-loading group developed AKI according to the SCr criteria, and none met the urine output criteria. In contrast, in the control group, staging was performed according to the SCr and urine output criteria in 19 (9.0%) and 4 (1.9%) patients, respectively; 3 (1.4%) patients fulfilled both criteria (p = 0.110). Of note, two patients in the control group required postoperative kidney replacement therapy, whereas none in the protein-loading group required such therapy. All 15 patients in the protein-loading group who developed AKI exhibited AKI reversal before hospital discharge, but only 21 of 26 patients in the control group who experienced AKI exhibited AKI reversal before hospital discharge (100% vs. 80.8%; p = 0.139). Summary statistics of all patients categorized by AKI are reported in the Additional file 1: Table S2. Those with AKI were more likely than those without AKI to be older; have a higher body mass index; have hypertension, diabetes mellitus, peripheral vasculopathy, and dyslipidemia; and have lower eGFR values at admission.

**Table 2:** Outcomes

	Control group (n = 214)	Protein-loading group (n = 110)	p-value (unadjusted)	p-value (adjusted)^a^
Serum creatinine, mg/dL^b^				
Hospital admission	0.85 (0.72 – 0.96)	0.84 (0.73 – 0.96)	0.998	0.930
Preoperative	0.84 (0.72 − 0.95)	0.83 (0.72 − 0.96)	0.956	0.883
Postoperative day 1	0.77 (0.65 − 0.89)	0.79 (0.70 − 0.88)	0.181	0.148
Postoperative day 2	0.84 (0.70 − 0.96)	0.82 (0.70 − 0.96)	0.308	0.251
Hospital discharge	0.8 (0.67 − 0.92)	0.78 (0.69 − 0.93)	0.990	0.924
3-month follow-up	0.90 (0.76 − 1.00)	0.83 (0.72 − 0.95)	0.088	0.048
1-year follow-up	0.95 (0.81 – 1.09)	0.87 (0.76 – 0.93)	< 0.001	< 0.001
eGFR, mL/min/1.73 m^2 c^				
Hospital admission	90 (80 − 98)	91 (82 − 100)	0.886	0.823
Preoperative	91 (80 − 98)	92 (82 − 100)	0.886	0.823
Hospital discharge	94 (85 − 102)	93 (85 − 102)	0.774	0.767
3-month follow-up	87 (77 − 95)	91 (82 − 100)	0.033	0.028
1-year follow-up	81 (70 − 88)	88 (78 − 96)	< 0.001	< 0.001
Albuminuria, mg/g creatinine				
Hospital admission	7.0 (3.6 − 24.1)	6.5 (3.3 − 21.0)	0.273	0.265
3-month follow-up	14.1 (4.3 − 62.1)	7.9 (1.6 − 43.9)	0.076	0.065
1-year follow-up	20.9 (4.3 − 62.1)	10.0 (1.8 − 26.0)	0.024	0.020
Postoperative day 1 urine output/hour, mL	2.7 (2 – 3.7)	2.33 (1.86 − 3.11)	0.021	0.031
Postoperative day 2 urine output/hour, mL	1.42 (1.13 – 1.72)	1.48 (1.2 − 1.87)	0.324	0.277
AKI, n (%)			0.945	0.945
Stage 1	18 (8.5%)	10 (9.1%)		
Stage 2	6 (2.8%)	4 (3.6%)		
Stage 3	2 (0.9%)	1 (0.9%)		
Postoperative kidney replacement therapy, n (%)	2 (0.9%)	0 (0%)	0.550	0.550
Type of AKI			0.110	0.110
Serum creatinine criteria	19 (9.0%)	15 (13.6%)		
Urine output criteria	4 (1.9%)	0 (0%)		
Both criteria	3 (1.4%)	0 (0%)		
AKI reversal state at discharge, n (%) ^d^			0.139	0.139
Reversal	21/26 (80.8%)	15/15 (100%)		
Non-reversal	5/26 (19.2%)	0 (0%)		

Summaries of quantitative variables are presented as median and interquartile range (in parentheses). For categorical variables, the absolute and relative frequencies (as %, in parentheses) are presented for the categories

*AKI* acute kidney injury; *eGFR* estimated glomerular filtration rate

^a^P-values were taken from linear models, including age, sex, body mass index, hypertension, and diabetes as covariables

^b^To convert the values for serum creatinine to micromoles per liter, multiply it by 88.4

^c^The eGFR was calculated using the Chronic Kidney Disease Epidemiology Collaboration equation [15]

^d^AKI reversal was defined as the absence of any stage of AKI based on either the serum creatinine or urine output criteria at hospital discharge [22]

### Changes in eGFR

**Fig. 1:**
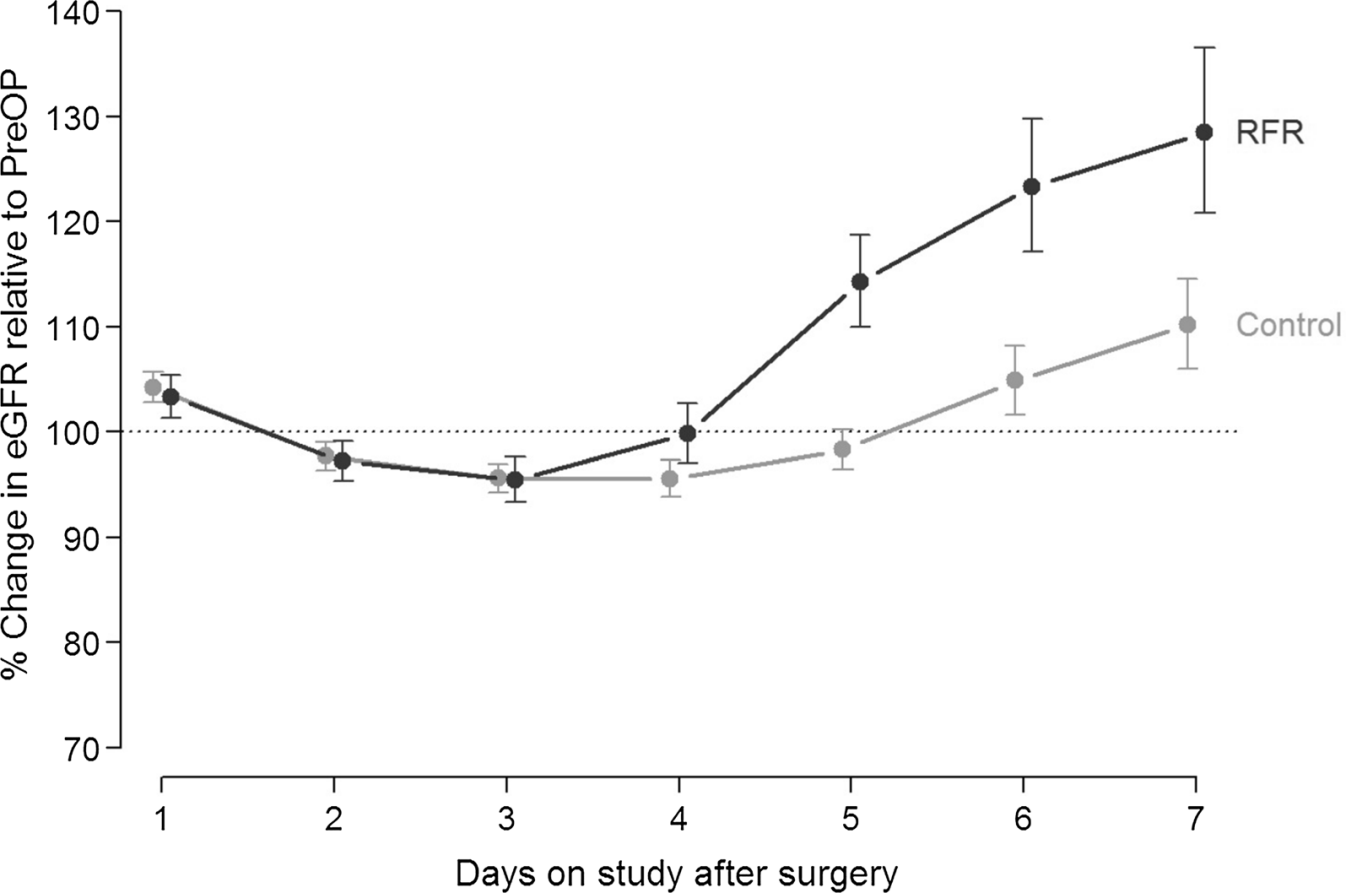
Changes in eGFR. eGFR by study day for patients receiving preoperative protein loading or standard preoperative care, change from preoperative values. p < 0.001 for the interaction day:group. Light grey: patients receiving standard preoperative care; dark grey: patients receiving preoperative high-oral protein load. The points are mean values with error bars, indicating the 95% confidence intervals. eGFR, estimated glomerular filtration rate

Over the first 7 days after surgery, mean eGFR started increasing on day 4. The increase was more pronounced in the protein-loading group than in the control group (p < 0.001; Fig. 1), reaching 120% of the preoperative value on day 7 after surgery.

**Fig. 2:**
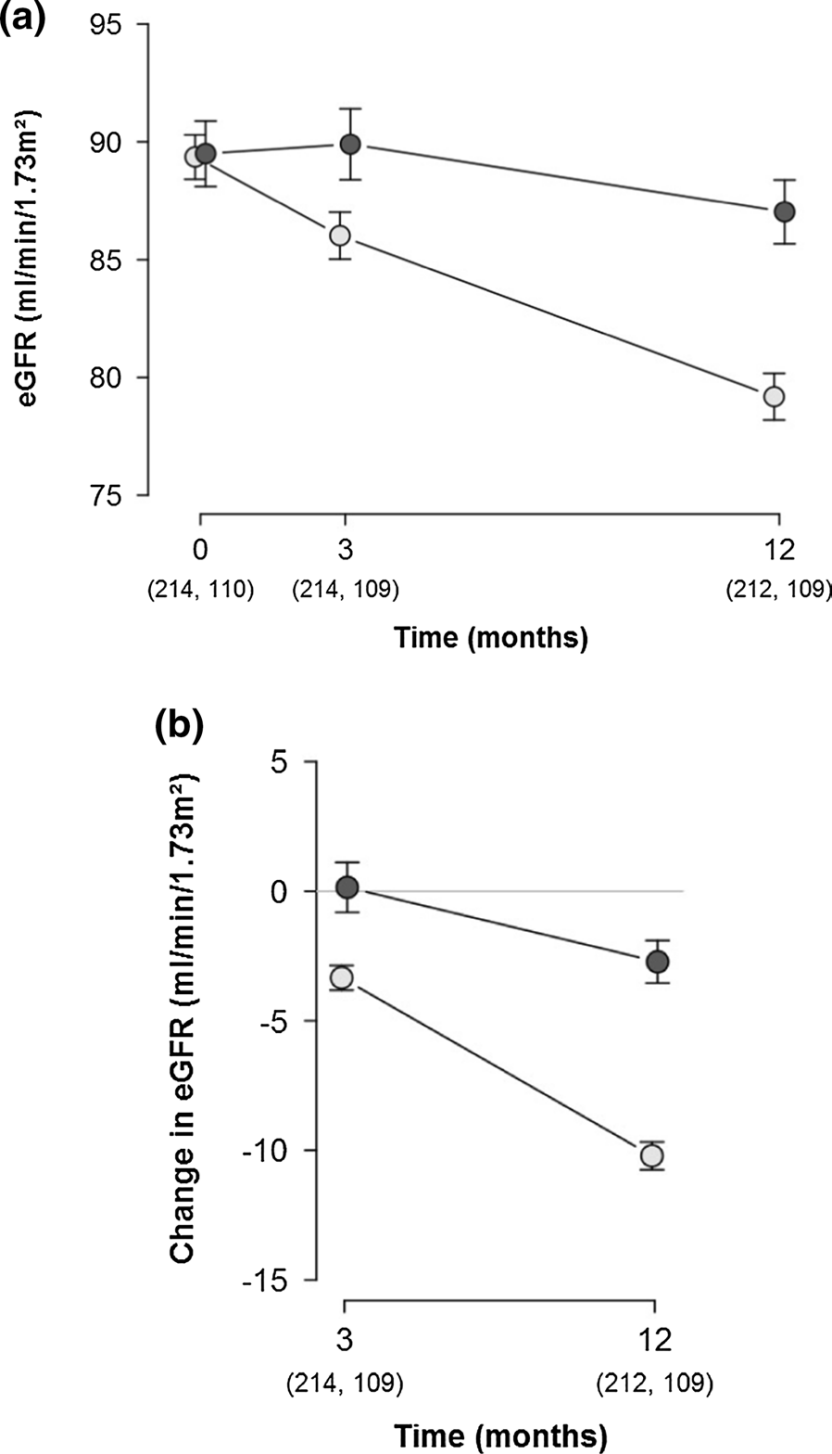
eGFR among patients receiving a high-oral protein load compared to those receiving standard preoperative care. **a** eGFR at the time of admission as well at 3 and 12 months after surgery. **b** Change in eGFR relative to the time of admission. Light grey: patients receiving standard preoperative care; dark grey: patients receiving preoperative high-oral protein load. The points are mean values with error-bars, indicating the 95% confidence intervals. The number of patients at different time points for each group are provided in parentheses. eGFR, estimated glomerular filtration rate

**Fig. 3:**
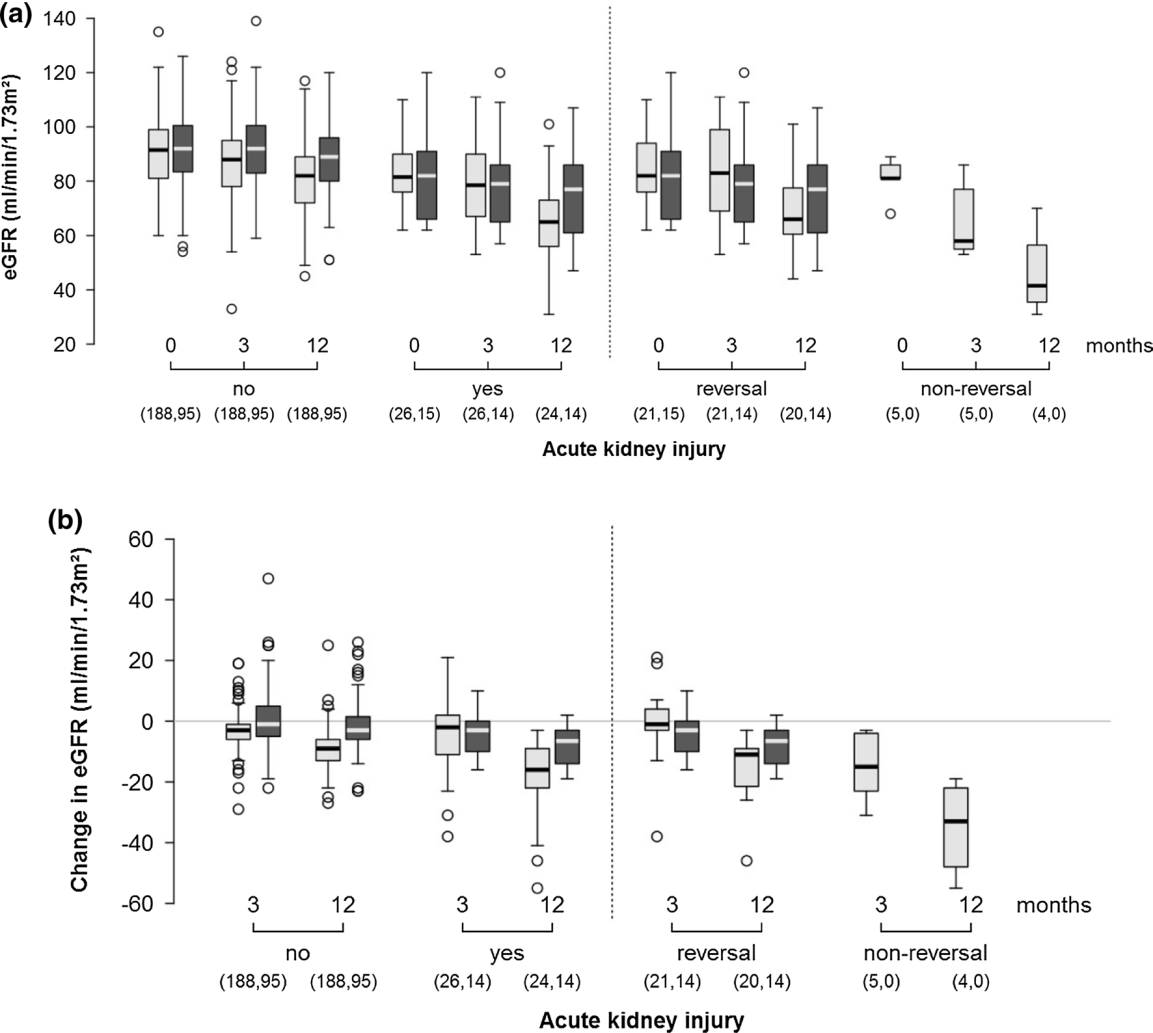
eGFR categorized on the basis of the occurrence of AKI and the AKI reversal status among patients receiving a preoperative high-oral protein load compared to those receiving standard preoperative care. **a** eGFR at the time of admission and at 3 and 12 months after surgery. **b** Changes in the eGFR relative to the time of admission. Light grey: patients with standard preoperative care; dark grey: patients receiving a preoperative high-oral protein load. The number of patients in each group at different time points are provided in parentheses. AKI, acute kidney injury; eGFR, estimated glomerular filtration rate

At hospital discharge, almost all patients (98.5%) had a normal renal function, as defined by the eGFR (≥ 60 mL/min/1.73 m^2^; p = 0.774). The eGFR values at 3 and 12 months after surgery in both groups are shown in Figs. 2a, 3a, and are listed in Table 3a. The changes in eGFR values, relative to the preoperative values, per patient are shown in Figs. 2b, 3b, and are listed in Table 3b. At both time points, the patients in the protein-loading group showed higher eGFR values than those in the control group (90 [95% CI 87–93] vs. 86 mL/min/1.73 m^2^ [95% CI 84–88], p = 0.028 at 3 months; and 87 [95% CI 84–90] vs. 79 mL/min/1.73 m^2^ [95% CI 77–81], p < 0.001 at 12 months). This effect was also visible among patients who did not develop AKI (91 [95% CI 89–94] vs. 87 mL/min/1.73 m^2^ [95% CI 85–89], p = 0.011 at 3 months; and 89 [95% CI 86–91] vs. 81 mL/min/1.73 m^2^ [95% CI 79–83], p < 0.001 at 12 months). The data for patients who developed AKI was insufficient to interpret the difference in eGFR levels between the groups. However, the mean decrease in eGFR levels per patient after 12 months was less pronounced in the protein-loading group for patients who developed AKI (− 8 [95% CI − 14 to − 1.9] vs. − 19 mL/min/1.73 m^2^ [95% CI − 23 to  − 14], p = 0.008 for all AKI cases; and − 8 [95% CI − 13 to  − 3.2] vs. − 15 mL/min/1.73 m^2^ [95% CI − 19 to  − 11], p = 0.022 for AKI reversal cases). Patients without AKI reversal exhibited the highest risk for long-term renal function loss, with a mean eGFR loss of –35.0 mL/min/1.73 m^2^ [95% CI − 60.9 to − 9.1]. Results remained significant after adjustment for potential confounders.

**Table 3:** eGFR among patients receiving high-oral protein loading, compared to that in patients receiving standard care prior to cardiac surgery

	Control group	Protein-loading group	Difference (protein load vs. control)	p-value (unadjusted)	p-value (adjusted)^b^
(a) eGFR values (in mL/min/1.73 m^2^)^a^					
3 months					
All patients (n = 323)	86 (84–88)	90 (87–93)	3.9 (0.42–7.3]	0.028	0.017
No AKI (n = 283)	87 (85–89)	91 [89–94)	4.6 (1.1–8.1)	0.011	0.005
AKI (n = 40)	80 (73–87)	79 (69–89)	0.0 (− 13 to 12)	0.936	0.619
Reversal (n = 35)	83 (75–91)	79 (70–89)	− 3.8 (− 16 to 8.7)	0.538	0.323
Non-reversal	66 (47–84)	–	–	–	
12 months					
All patients (n = 321)	79 (77–81)	87 (84–90)	7.8 (4.5–11)	< 0.001	< 0.001
No AKI (n = 283)	81 (79–83)	89 (86–91)	7.9 (4.6–11)	< 0.001	< 0.001
AKI (n = 38)	65 (58–72)	75 (66–84)	9.6 (− 1.7 to 21)	0.093	0.619
Reversal^c^ (n = 34)	69 (62–76)	75 (67–83)	5.8 (− 4.9 to 17)	0.280	0.316
Non-reversal	46 (19–73]	–	–	–	
(b) Change in eGFR (in mL/min/1.73 m^2^)^a^ relative to the preoperative values					
3 months					
All patients (n = 323)	− 3.3 (− 4.4 to − 2.2)	0.15 (− 1.4 to 1.7)	3.5 (1.6 to 5.4)	< 0.001	< 0.001
No AKI (n = 283)	− 3.2 (− 4.3 to − 2.1)	0.68 (− 0.87 to 2.2]	3.9 (2.0 to 5.8)	< 0.001	< 0.001
AKI (n = 40)	− 4.2 (− 8.7– 0.44)	− 3.5 (− 9.8 to 2.8)	0.65 (− 7.1 to 8.4)	0.865	0.817
Reversal^c^ (n = 35)	− 1.5 (− 6.2 to 3.2)	− 3.5 (− 9.3 to 2.3)	− 2.0 (− 9.4 to 5.5)	0.594	0.764
Non-reversal	− 15 (− 30 to − 0.2)	–	–	–	
12 months					
All patients (n = 321)	− 10 (− 11 to − 9.1)	− 2.7 (− 4.2 to − 1.2)	7.5 (5.6 to 9.4)	< 0.001	< 0.001
No AKI (n = 283)	− 9.1 (− 10 to − 8.1)	− 1.9 (− 3.4 to − 0.52)	7.2 (5.4 to 8.9)	< 0.001	< 0.001
AKI (n = 38)	− 19 (− 23 to − 14)	− 8 (− 14 to − 1.9]	11 (3 to 18)	0.008	0.011
Reversal^c^ (n = 34)	− 15 (− 19 to − 11)	− 8 (− 13 to − 3.2)	7.3 (1.1 to 14)	0.022	0.028
Non-reversal	− 35 (− 61 to − 9.1)	–	–	–	

Summaries of quantitative variables are presented as mean and 95% confidence interval (in brackets)

*AKI* acute kidney injury; *eGFR* estimated glomerular filtration rate

^a^The eGFR was calculated using the Chronic Kidney Disease Epidemiology Collaboration equation [15]

^b^P-values were taken from linear models, including age, sex, body mass index, hypertension, and diabetes as covariables

^c^Reversal after AKI was defined as the absence of any stage of AKI based on either the serum creatinine or urine output criteria at hospital discharge [22]

Over the 12-month follow-up period, the mean eGFR change per month in the protein-loading group was − 0.23 mL/min/1.73 m^2^ [95% CI − 0.54 to  − 0.08], as compared to − 0.83 mL/min/1.73 m^2^ [95% CI − 1.05– − 0.61] in the control group. The mean difference in these slopes was 0.60 mL/min/1.73 m^2^ ([95% CI 0.22–0.97]; p = 0.002; Fig. 2a). The changes in SCr are described in the Additional file 1; Fig. S4.

### Changes in albuminuria

There were no detectable differences in albuminuria between the protein loading vs. the control group at hospital admission (29.7 mg/g [95% CI 10.7–48.6] vs. 32.6 [95% CI 12.1–54.2], p = 0.457) and at 3 months after surgery (115.1 mg/g [95% CI 26.3–203.9] vs. 165.2 mg/g [95% CI 34.7–267.7], p = 0.063). However, patients in the protein loading group as compared to the control group had lower levels of albuminuria at 12 months after surgery (125.3 mg/g [95% CI 25.5–225.3] vs. 193.8 mg/g [95% CI 26.4–302.4], p = 0.015). The result remained significant after adjustment for potential confounders.

### TIMP-2 and IGFBP7 secretion in in vitro model systems

**Fig. 4:**
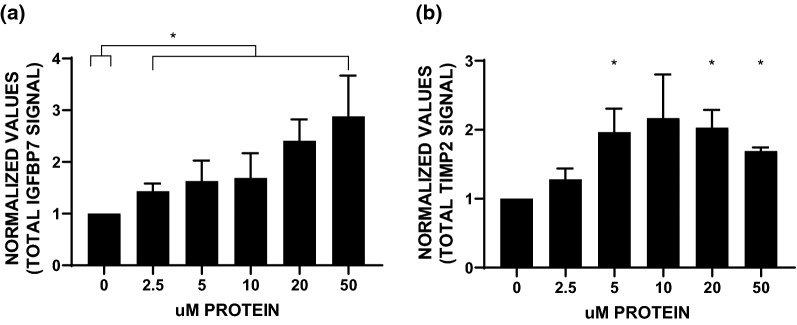
Effect of dietary protein dose response treatment of primary human kidney tubule cells on the presence of TIMP-2 and IGFBP7 in the apical conditioned media. Isolated primary human proximal and distal tubule cells were subjected to dose–response analysis with whey protein, and secretion of the AKI biomarkers IGFBP7 and TIMP2 was assessed by immunoblot analysis of apical conditioned media. Densitometry values for (**A**) IGFBP7 from proximal tubule cells (4 genetically different samples) and (**B**) TIMP-2 from distal tubule cells (3 genetically different samples) were adjusted to protein concentration and normalized to no treatment. Asterisks indicate statistical significance from no treatment (p < 0.05). AKI, acute kidney injury; IGFBP7, insulin-like growth factor binding protein 7; TIMP-2, tissue inhibitor of metalloproteases-2

We next assessed whether a high protein load could affect kidney tubule cells directly using an in vitro cell culture model system to determine whether protein administration mediates the release of TIMP-2 and IGFBP7. We had previously identified that IGFBP7 secretion is primarily a proximal tubule phenomenon, and TIMP2 expression and secretion are distal tubule phenomena [23]; therefore, biomarker secretion was assessed in the appropriate systems with increasing doses of exogenous protein (Fig. 4). This analysis identified that application of dietary protein directly onto kidney tubule epithelial cells of proximal and distal tubule origin is indeed capable of inducing a statistically significant increase in the amounts of both biomarkers in the apical (luminal) conditioned media.

## Discussion

### Key findings

In patients with presurgical eGFR values ≥ 60 mL/min/1.73 m^2^ undergoing cardiac surgery, a preoperative high-oral protein load did not reduce the occurrence of perioperative AKI, but was associated with an improved eGFR postoperatively and more preserved eGFR at 3 and 12 months after surgery, even after stratification based on the occurrence of AKI. Additionally, we validated an experimental proof of concept in vitro that direct interaction of dietary protein with kidney tubule cells can increase the amounts of biomarkers implicated in cell cycle arrest and tubule cell protection in apical media.

### Relationship to previous studies

Protein intake reportedly increases renal blood flow and GFR in healthy humans, and at any given level of protein intake, GFR is greater in younger than in older individuals and greater in women and men [26]. Although patients with CKD are likely to exhibit progression to advanced stages more rapidly if they consume a high-protein diet over a prolonged period [27, 28], consistent with the hypothesis that the glomerular hemodynamic changes associated with persistent hyperfiltration accelerate glomerular injury [29], a short-term high-protein diet may induce reno-protective and restorative mechanisms. This hypothesis is based on animal models, in which protein loading was shown to protect the kidneys from ischemic insults when administered before the harmful exposure [11]. The specific mechanisms remain unclear but appear to involve effects beyond an increase in renal blood flow, since selective agents have not been effective at preserving GFR post-cardiac surgery [5]. The additional direct effects of a high protein load on kidney tubular cells—the cells most affected in AKI—remains unclear.

Proteins, once filtered at the glomerular level, are normally almost entirely reabsorbed at the proximal tubular level [30]. Experimental studies indicate that protein overload of tubule cells may elicit tubular epithelial injury and peritubular inflammation through upregulation of inflammatory and fibrogenic genes and production of related proteins [31–33]. Further, apoptosis and autophagy are other mechanisms that underlie protein-induced tubular cell injury [34, 35]. As such, protein overload has been reported to cause a dose- and duration-dependent induction of apoptosis in cultured proximal tubule cells [34].

However, the protein loads administered in this study were well within normal dietary variations and were not likely to induce damage to kidney cells as described above. Nevertheless, we speculate that these levels may have “stressed” the kidney in a manner analogous to preconditioning with ischemia [36]. Zarbock and coworkers recently showed that the protective effects of RIPC are dependent on eliciting a kidney stress response quantified by urinary biomarkers TIMP-2 and IGFBP7 [12, 13]. While these same biomarkers have been shown to predict AKI after cardiac surgery [37, 38], their release in response to sub-injurious noxious stimuli may invoke a protective response.

Mechanistically, TIMP-2 and IGFBP7 are two independent proteins that are released in response to proximal and distal tubular insults and are thought to be engaged in self-protective mechanisms such as AKI-induced transient cell cycle arrest [37, 39, 40]. A recent trial involving patients undergoing cardiac surgery demonstrated that preoperative RIPC reduced postoperative AKI [12]. Moreover, the effectiveness of this intervention was dependent on the release of TIMP-2 and IGFBP7 since no protection was observed in a patient who did not respond to RIPC with an increase in TIMP-2 and IGFBP7 before surgery. Conversely, patients exhibiting this response showed a reduced rate of AKI. These findings substantiated our hypothesis that the release of TIMP-2 and IGFBP7 elicited by sub-injurious stimuli (e.g., brief ischemic preconditioning) may indicate an adaptive and desensitizing response of kidney tubule cells to subsequent inflammatory and ischemic injury [41]. On the basis of our experimental model, we speculate that a short-term high dietary protein load in patients with normal renal function may act similar to ischemic preconditioning by inducing a protective response through the release of TIMP-2 and IGFBP7.

### Study strengths and limitations

In line with a recent pilot study [42], our results suggest that a preoperative high-oral protein load may influence renal outcomes post-cardiac surgery, even though it does not reduce the occurrence of perioperative AKI. Consistent with previous publications [25], our findings emphasize the incorporation of longer follow-up periods to determine the effects of any intervention on renal function beyond a simple reduction in perioperative AKI. Other strengths of the study include SCr measurements under rigorous conditions, the use of a blinded adjudication committee for renal outcome measure assessments, suspension of renin–angiotensin-system blockers prior to surgery and outpatient visits, the low dropout rate, and the provision of an experimental proof of concept of a potential mechanism of action of positive long-term outcomes. Notably, the in vitro work was intentionally designed to be a simple proof of concept experiment to identify whether or not protein load can have a direct effect on kidney tubule cells and biomarker presence in the conditioned media/urine. Further studies are necessary to elucidate the molecular mechanisms of action by which high-oral protein loading may induce kidney-protective effects (including techniques for specific blockage). TIMP-2 and IGFBP7 were chosen as biomarkers of interest as both are one of the few markers potentially implicated in kidney protection as opposed to kidney damage (i.e., neutrophil gelatinase-associated lipocalin and liver-type fatty acid binding protein). It is noteworthy that the different trends of both biomarkers following increasing doses of exogenous protein was not unexpected. Indeed, a significant finding in our initial publication on IGFBP7 and TIMP-2 in human kidney cells [23] was that the biomarkers primarily originate from different regions of the nephron, where IGFBP7 is primarily secreted by proximal tubule cells, and TIMP-2 is expressed and secreted only from cells of distal tubule origin. Given that proximal and distal tubule cells are differentially affected by insults, different molecules at different concentrations and/or temporal modulations are likely to be used to induce the response necessary for the protection of that cell type (e.g. cell cycle arrest). Secondly, while the arithmetic product of these biomarkers ([TIMP-2]•[IGFBP7]) was identified as the best for the prediction of the onset of AKI in the critically ill patient [37], there is no clear biological association between the two. While both can affect cell cycle arrest, IGFBP7 is involved in the insulin signaling nexus [43], and TIMP-2 is involved in the extracellular matrix remodeling nexus [44], and there is currently no known biological association between both biomarkers that would suggest an expectation that their secretion profiles should behave similarly. Lastly, while both biomarkers together provide the most specific prediction of AKI, each biomarker alone can be more predictive in different etiologies of AKI (e.g., sepsis versus cardiac surgery), demonstrating that the secretion profiles of each biomarker are differentially affected by different effector molecules specific for each etiology.

Our findings may have important implications for clinical practice. First, recent evidence has shown that the AKI risk after cardiac surgery is potentially modifiable [45]. However, this finding was determined on the basis of early postoperative biomarker assessment, which identified high-risk patients and linked them to a “care bundle”. Although this strategy is effective, it requires careful coordination between biomarker measurement and postoperative care. If our results can be confirmed by trials adequately powered to detect improvements in renal outcome, a preoperative high-protein diet may represent a simple and cost-efficient treatment for preserving long-term GFR. Certainly, a preoperative treatment with high-oral protein load may be more convenient and easier to replicate compared to a treatment with intravenous amino acid infusion. Further studies are needed to investigate the mechanisms underlying the potential protective effects of short-term protein loading on renal function.

Our study had limitations, including its non-blinded, non-randomized, and single-center design as well as the relatively low AKI rate. However, the measures of renal function were based on objective assays conducted by a central laboratory unaware of the group allocation. Moreover, the low occurrence of AKI was expected since we excluded patients with reduced eGFR, and selected patients with low comorbidities, elective surgical procedures, and a high rate of minimally invasive surgery; all factors associated with low AKI rates [46]. Besides, our results should be interpreted with caution, since estimates of renal function solely based on SCr may lead to overestimation of GFR [47]. We did not have information on the food composition of standard preoperative care provided in the cardiac surgery unit. However, given that both groups received the same standard preoperative care, it is unlikely that the composition had a major effect on the observed results. Furthemore, the amount of protein provided in the form of cooked meat prior to surgery may be stressful for some patients and cumbersome to implement into clinical practice. Thus, alternative more feasible preparations for protein loading should be investigated (e.g., using protein powder). Surrogate markers for estimation of protein intake (e.g., using the Maroni’s method) may be considered in future trials. Moreover, patients in the protein loading vs. the control group required more perioperative red blood cell transfusion, which may have influenced the results. It should be noted, however, that the need for perioperative red blood cell transfusion did not differ when categorized by the occurrence of AKI. Finally, we did not have information on diet and medication between the both groups during the first year after surgery except on the use of renin–angiotensin-system blockers, which remain to be vital confounding factors for the final results.

## Conclusions

In summary, among patients undergoing cardiac surgery, a preoperative high-oral protein load did not reduce the occurrence of AKI (in comparison with standard care) but was associated with an improved eGFR postoperatively and more preserved eGFR at 12 months, even after stratification based on the occurrence of AKI. The potential mechanisms of action by which short-term protein loading induces a kidney-protective response may include the maintenance of renal blood flow and cell cycle inhibition of renal tubular epithelial cells. The observed preservation of renal function in the protein-loading group warrants further investigation.

## Supplementary Information

**Additional file 1: Figure S1.** Study flow chart. The diagram describes the protocol used for the enrolment of patients in the present study. ACEis, angiotensin-converting-enzyme inhibitors; ARB, angiotensin II receptor blockers; IV, intravenous; NSAIDs, non-steroidal anti-inflammatory drugs. **Figure S2.** Changes in eGFR among patients receiving high oral protein loading compared to those receiving standard care prior to cardiac surgery. The figure illustrates the changes in eGFR among patients receiving preoperative high oral protein loading (dark-grey circles), as compared to those in patients receiving standard care (light-grey circles) at different time points (a) and compared to the preoperative values (b). Horizontal lines indicate median values, boxes indicate the inter-quartile range, and whiskers indicate the minimum and maximum values. Data beyond the whiskers are plotted as outliers (circles). The number of patients in each group at different time points for each group are presented in parentheses. eGFR, estimated glomerular filtration rate. **Figure S3.** Serum creatinine levels among patients receiving high oral protein loading compared to those receiving standard preoperative care prior to cardiac surgery. (a) Serum creatinine levels at the time of admission as well at 3 and 12 months after surgery. (b) Change in serum creatinine levels relative to the time of admission. Light grey: patients with standard preoperative care; dark grey: patients receiving preoperative high oral protein loading. Points are mean values with error bars indicating the 95% confidence intervals. The number of patients in each group at different time points for each group are presented in parentheses. **Figure S4.** Serum creatinine levels categorized by the occurrence of AKI and AKI reversal at hospital discharge among patients receiving preoperative high oral protein loading compared to those receiving standard preoperative care. (a) Serum creatinine levels at the time of admission as well at 3 and 12 months after surgery. (b) Change in serum creatinine levels, relative to the time of admission. Light grey: patients with standard preoperative care; dark grey: patients receiving preoperative high oral protein loading. Number of patients at different time points for each group are presented in parentheses. AKI, acute kidney injury. **Table S1.** Bioimpedance vector analysis of the protein loading group categorized by occurrence of postoperative AKI. **Table S2.** Baseline, operative, ICU and follow-up characteristics categorized by occurrence of AKI. **Table S3.** Serum creatinine levels among patients receiving high oral protein loading, as compared to those in patients receiving standard care prior to cardiac surgery.

## Data Availability

The datasets used and/or analyzed during the study are available from the corresponding author upon reasonable request.

## Abbreviations

AKI: Acute kidney injury
CKD: Chronic kidney disease
GFR: Glomerular filtration rate
eGFR: Estimated glomerular filtration rate
IGFBP7: Insulin-like growth factor–binding protein 7
RIPC: Remote ischemic preconditioning
SCr: Serum creatinine
TIMP-2: Tissue inhibitor of metalloproteases-2
