# Supplementary material for: Effects of preoperative high-oral protein loading on short- and long-term renal outcomes following cardiac surgery: a cohort study
Source: J Transl Med. 2022 May 10;20:204. doi: 10.1186/s12967-022-03410-x (PMC9092825; doi:10.1186/s12967-022-03410-x)
Supplement: Supplementary file 1 — Additional file 1: Figure S1. Study flow chart. The diagram describes the protocol used for the enrolment of patients in the present study. ACEis, angiotensin-converting-enzyme inhibitors; ARB, angiotensin II receptor blockers; IV, intravenous; NSAIDs, non-steroidal anti-inflammatory drugs. Figure S2. Changes in eGFR among patients receiving high oral protein loading compared to those receiving standard care prior to cardiac surgery. The figure illustrates the changes in eGFR among patients receiving preoperative high oral protein loading (dark-grey circles), as compared to those in patients receiving standard care (light-grey circles) at different time points (a) and compared to the preoperative values (b). Horizontal lines indicate median values, boxes indicate the inter-quartile range, and whiskers indicate the minimum and maximum values. Data beyond the whiskers are plotted as outliers (circles). The number of patients in each group at different time points for each group are presented in parentheses. eGFR, estimated glomerular filtration rate. Figure S3. Serum creatinine levels among patients receiving high oral protein loading compared to those receiving standard preoperative care prior to cardiac surgery. (a) Serum creatinine levels at the time of admission as well at 3 and 12 months after surgery. (b) Change in serum creatinine levels relative to the time of admission. Light grey: patients with standard preoperative care; dark grey: patients receiving preoperative high oral protein loading. Points are mean values with error bars indicating the 95% confidence intervals. The number of patients in each group at different time points for each group are presented in parentheses. Figure S4. Serum creatinine levels categorized by the occurrence of AKI and AKI reversal at hospital discharge among patients receiving preoperative high oral protein loading compared to those receiving standard preoperative care. (a) Serum creatinine levels at the time of admission as we [file 12967_2022_3410_MOESM1_ESM.docx]

**SUPPLEMENTARY MATERIAL**

**Effects of preoperative high oral protein loading on short- and long-term renal outcomes following cardiac surgery: a cohort study**

Faeq Husain-Syed MD, David R. Emlet PhD, Jochen Wilhelm PhD, Tommaso Hinna Danesi MD, Fiorenza Ferrari MD, Pércia Bezerra MD, Salvador Lopez-Giacoman MD, Gianluca Villa MD, Khodr Tello MD, Horst-Walter Birk MD, Werner Seeger MD, Davide Giavarina MD, Loris Salvador MD, Dana Y. Fuhrman DO, MS, John A. Kellum MD, Claudio Ronco MD and the IRRIV-AKI Study Group

Members of the IRRIV-AKI Study Group: Carlotta Caprara MD, Valentina Corradi MD, Massimo de Cal MD, Carla Estremadoyro MD, Renhua Lu MD, Sara Samoni MD PhD, Aashish Sharma MD, Lorenzo Tofani MD, Grazia Maria Virzì MD

This supplementary material has been provided by the authors to give readers additional information about their work.

**TABLE OF CONTENTS**

[SUPPLEMENTARY METHODS 3](#_Toc91825563)

[Patient selection criteria 3](#_Toc91825564)

[Procedures and measurements 3](#_Toc91825565)

[*Surgical procedures* 3](#_Toc91825566)

[*Evaluation of kidney function* 4](#_Toc91825567)

[*Other measures* 4](#_Toc91825568)

[*Sample collection and laboratory methods* 4](#_Toc91825569)

[*Amino acid profile used in in vitro cells culture model system* 4](#_Toc91825570)

[SUPPLEMENTARY FIGURES 6](#_Toc91825571)

[Figure S1: Study flow chart. 6](#_Toc91825572)

[Figure S2: Changes in eGFR among patients receiving high oral protein loading compared to those receiving standard care prior to cardiac surgery. 7](#_Toc91825573)

[Figure S3: Serum creatinine levels among patients receiving high oral protein loading compared to those receiving standard preoperative care prior to cardiac surgery. 10](#_Toc91825574)

[Figure S4: Serum creatinine levels categorized by the occurrence of AKI and AKI reversal at hospital discharge among patients receiving preoperative high oral protein loading compared to those receiving standard preoperative care. 11](#_Toc91825575)

[SUPPLEMENTARY TABLES 12](#_Toc91825576)

[Table S1: Bioimpedance vector analysis of the protein loading group categorized by occurrence of postoperative AKI 12](#_Toc91825577)

[Table S2: Baseline, operative, ICU and follow-up characteristics categorized by occurrence of AKI 13](#_Toc91825578)

[Table S3: Serum creatinine levels among patients receiving high oral protein loading, as compared to those in patients receiving standard care prior to cardiac surgery 16](#_Toc91825579)

[SUPPLEMENTARY REFERENCES 18](#_Toc91825580)

#

# SUPPLEMENTARY METHODS

## Patient selection criteria

The selection criteria of the study *‘Preoperative Renal Functional Reserve Predicts Risk of Acute Kidney Injury* after Cardiac Operation’ [1] were used to enroll patients for the control group.

The following inclusion criteria were applied: subjects aged >18 years undergoing elective cardiac surgery and those with an estimated glomerular filtration rate (eGFR) >60 mL/min/1.73 m^2^ using the Chronic Kidney Disease (CKD) Epidemiology Collaboration equation [2] who were scheduled to undergo elective cardiac surgery (coronary artery bypass, valve replacement, a combination of the two, or other surgery, with cardiopulmonary bypass).

The following patients were excluded: who did not discontinue treatment with angiotensin-converting-enzyme inhibitors or angiotensin II receptor blockers (part of the standard procedure at the Department of Cardiac Surgery in Vicenza), received non-steroidal anti-inflammatory drugs within a minimum of 48 h before admission, or were administered intravenous contrast media within the 72 h before hospital admission. Other exclusion criteria were as follows: Pre-existing acute kidney injury (AKI) [3], estimated glomerular filtration rate (eGFR) values <60 mL/min/1.73 m^2^, solitary kidney, diabetes mellitus type 1, recent cardiac arrest or myocardial infarction up to 7 days before surgery, liver failure or cirrhosis, total parenteral nutrition, pregnancy, hemoglobin levels <11 g/dl, sepsis, history of malabsorption (e.g., chronic inflammatory bowel disease, short bowel, or pancreatic insufficiency), transplant donor or recipient, active autoimmune disease with renal involvement, rhabdomyolysis, prostate hypertrophy with International Prostate Symptom Score ≥20, and neoplasm.

All patients included in the study were to be followed until the scheduled end of the study. Data collection could be terminated prior to the scheduled time only under the following conditions:

- A patient or his/her legal representative may refuse further participation in the study at any time (withdrawal of consent).
- The investigator may withdraw a patient from the participation in the study at any time because of a severe protocol violation, or the development of incidents/near-incidents/other severe clinical complications related to the study protocol.

In both the protein load and standard care groups, the attending clinician determined all aspects of preoperative, intraoperative, and postoperative care, including the anesthetic regimen, cardiopulmonary bypass parameters, fluid replacement, and nutrition support based on their standard practice and individual patient’s needs, independently of the study group.

## Procedures and measurements

### *Surgical procedures*

Cardiac surgery was performed as conventional sternotomic or minimally invasive cardiac surgery. Conventional sternotomic approach was the standard of care for a) isolated coronary artery bypass graft; b) coronary artery bypass graft combined with other surgeries (valvular surgery, maze procedure, replacement of the ascending aorta or surgery of atrial septal defects and cardiac tumors); and c) replacement of the ascending aorta with or without valve surgery. Minimally invasive surgery included both mini-sternotomic and endoscopic surgical approaches. The endoscopic surgical approach was the standard of care for mitral valve and tricuspid valve surgeries, and surgery of atrial septal defects and cardiac tumors with no patient selection and without additional preoperative examinations. 60% of aortic valve surgeries were performed with endoscopic surgical approach; the other 40% were performed with a mini-sternotomic approach. Regardless the surgery, the standard setup for the minimally invasive group included one 3 cm working port (periareolar incision for mitral and tricuspid valve surgery, and atrial septum defect surgery; prepectoral incision for isolated aortic valve or combined aortic and other valve surgery) and two 5 mm miniports for the introduction of the 30° thoracoscope and the left ventricle vent-line. In all patients undergoing minimally invasive surgery, cardiopulmonary bypass was achieved from femoral vessels with retrograde perfusion.

### *Other measures*

Clinical variables were extracted from patients’ medical records. Kidney replacement therapy was administered based on the discretion of the treating physician. Complications were recorded, and perioperative myocardial infarction and stroke were defined as previously described [4].

Bioimpedance vector analysis (BIVA) was performed by using tetrapolar impedance plethysmography (EFG ElectroFluidGraph, Akern SRL, Florence, Italy) prior to protein loading (see Supplementary Table S1 for results). The following bioelectrical impedance vector analysis parameters were collected: resistance, reactance, phase angle, and hydration percentage of lean body mass. The bioelectrical parameters of resistance and reactance were measured using an alternating electric flow of 300 microA and an operating frequency of 50 kHz. Bioelectrical impedance vector analyses were performed by four different trained operators, with the patient in the supine position on the hospital bed and not touching any metal objects. The angles between the upper limbs and trunk and between the legs were 30° and 45°, respectively, as per the manufacturer’s indications. The skin was cleansed with alcohol or saline before the electrodes were applied to the right hand and foot. The normal level of hydration was set between 72.7% and 74.3% of lean body mass (class 0) [5].

### *Sample collection and laboratory methods*

In both groups, SCr was measured at hospital admission (before possible protein loading); immediately before surgery (to detect possible changes due to the creatinine content of the red meat); upon admission to the intensive care unit; daily for up to 7 days after surgery, per the discretion of the treating physician; at hospital discharge; and at follow-up 3 and 12 months post-surgery. All blood samples were analyzed at the local Department of Laboratory Medicine by technicians blinded to the clinical data. SCr was measured by the enzymatic method (IL test^TM^, Instrumentation^®^ Laboratory SpA, Milano, Italy) using an ILab650 analyzer (Instrumentation Laboratory, Werfen Group, Barcelona, Spain). Albuminuria was measured by a turbidimetric method on the ADVIA XPT analyzer, and normalized to the urinary creatinine concentration to account for urine dilution.

### *Amino acid profile used in in vitro cells culture model system*

Life Extension, Wellness Code® Whey Protein Concentrate

**Composition** **in 20 g**

Alanine  1.08 g

Arginine  0.56 g

Aspartic Acid  2.26 g

Cysteine  0.58 g

Glutamic Acid  3.72 g

Glycine  0.42 g

Histidine  0.40 g

Isoleucine 1.40 g

Leucine  2.28 g

Lysine  1.88 g

Methionine  0.52 g

Phenylalanine  0.70 g

Proline  1.30 g

Serine  1.08 g

Threonine  1.48 g

Tryptophan 0.42 g

Tyrosine 0.70 g

Valine  1.28 g

# SUPPLEMENTARY FIGURES

## Figure S1: Study flow chart.

The diagram describes the protocol used for the enrolment of patients in the present study.

ACEis, angiotensin-converting-enzyme inhibitors; ARB, angiotensin II receptor blockers; IV, intravenous; NSAIDs, non-steroidal anti-inflammatory drugs.

**(a)**


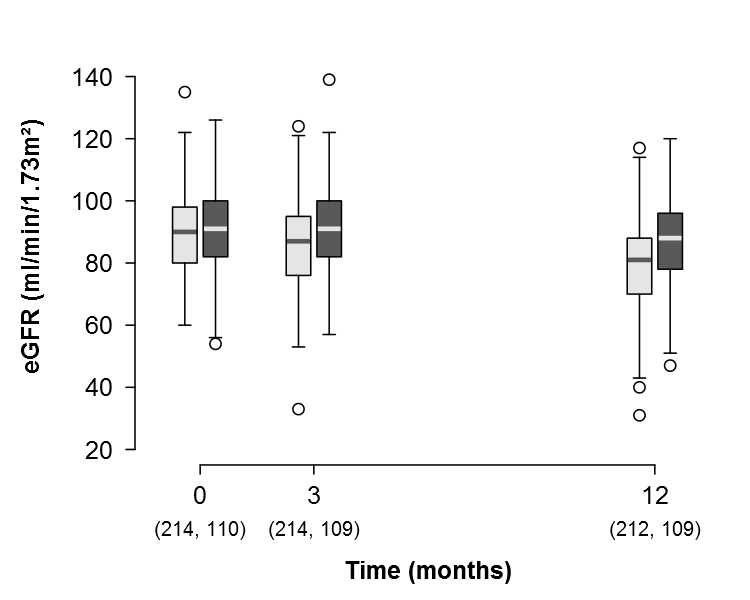


**(b)**


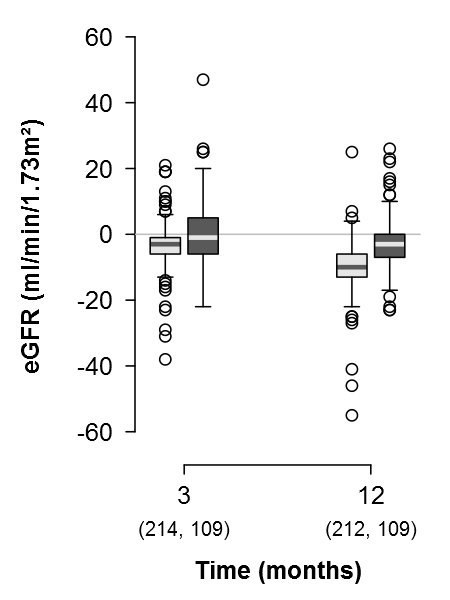


## Figure S2: Changes in eGFR among patients receiving high oral protein loading compared to those receiving standard care prior to cardiac surgery.

The figure illustrates the changes in eGFR among patients receiving preoperative high oral protein loading (dark-grey circles), as compared to those in patients receiving standard care (light-grey circles) at different time points **(a)** and compared to the preoperative values **(b)**. Horizontal lines indicate median values, boxes indicate the inter-quartile range, and whiskers indicate the minimum and maximum values. Data beyond the whiskers are plotted as outliers (circles). The number of patients in each group at different time points for each group are presented in parentheses.

eGFR, estimated glomerular filtration rate.

(a)


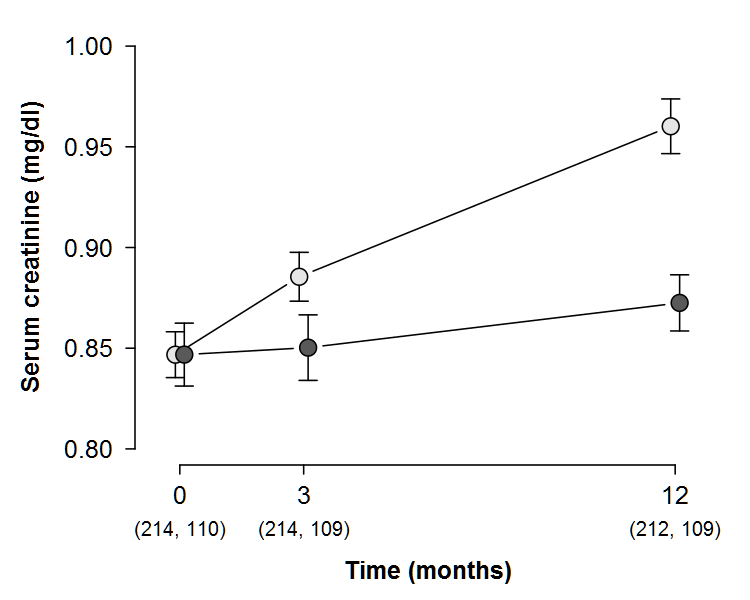


(b)


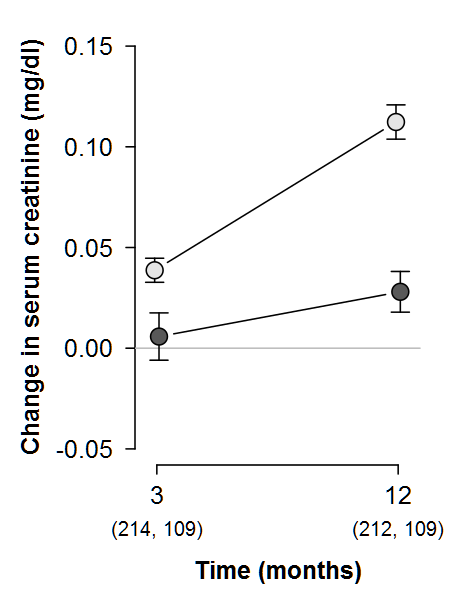


## Figure S3: Serum creatinine levels among patients receiving high oral protein loading compared to those receiving standard preoperative care prior to cardiac surgery.

**(a)** Serum creatinine levels at the time of admission as well at 3 and 12 months after surgery. **(b)** Change in serum creatinine levels relative to the time of admission. Light grey: patients with standard preoperative care; dark grey: patients receiving preoperative high oral protein loading. Points are mean values with error bars indicating the 95% confidence intervals. The number of patients in each group at different time points for each group are presented in parentheses.

**(a)**

**
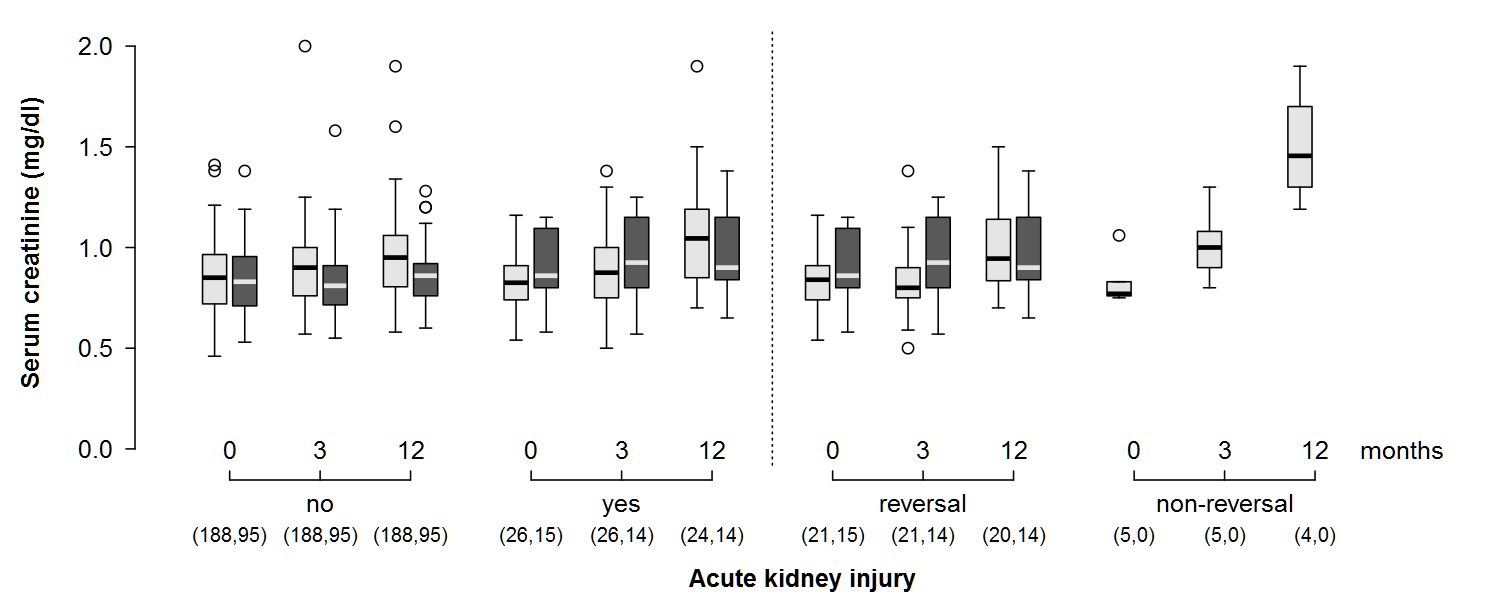
**

**(b)**

**
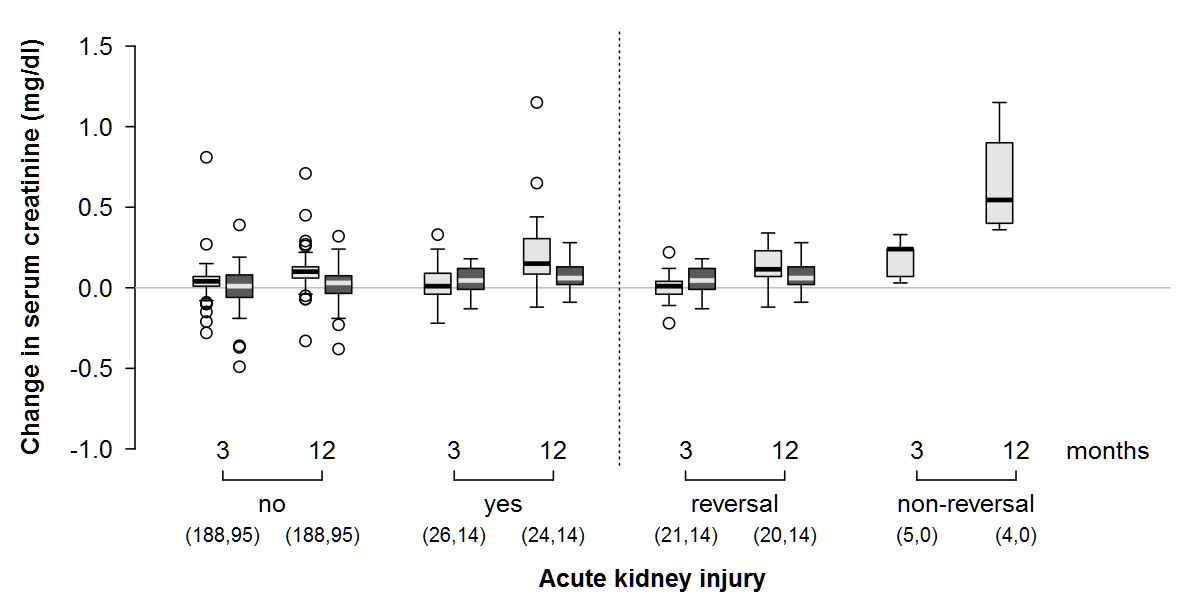
**

## Figure S4: Serum creatinine levels categorized by the occurrence of AKI and AKI reversal at hospital discharge among patients receiving preoperative high oral protein loading compared to those receiving standard preoperative care.

**(a)** Serum creatinine levels at the time of admission as well at 3 and 12 months after surgery. **(b)** Change in serum creatinine levels, relative to the time of admission. Light grey: patients with standard preoperative care; dark grey: patients receiving preoperative high oral protein loading. Number of patients at different time points for each group are presented in parentheses.

AKI, acute kidney injury.

# SUPPLEMENTARY TABLES

## Table S1: Bioimpedance vector analysis of the protein loading group categorized by occurrence of postoperative AKI

|  | **All patients**  **(n = 110)** | **No AKI**  **(n = 95)** | **AKI stage 1**  **(n = 10)** | **AKI stage 2-3**  **(n = 5)** |
| --- | --- | --- | --- | --- |
| BIVA evaluation prior to protein loading, % | 73.6 (73.3−73.8) | 73.6 (73.3−73.8) | 73.6 (73.3−73.9) | 73.6 (73.5−73.7) |

From Husain-Syed et al [1]. Variables are presented as median and interquartile range (in parentheses).

AKI, acute kidney injury; BIVA, bioimpedance vector analysis.

## Table S2: Baseline, operative, ICU and follow-up characteristics categorized by occurrence of AKI

|  | **No AKI**^†^  **(n = 283)** | **AKI**^†^  **(n = 41)** | **p-value** |
| --- | --- | --- | --- |
| **Demographics** |  |  |  |
| Age, years | 62 (53–70) | 71 (60–95) | 0.001 |
| Male sex, n (%) | 205 (72%) | 24 (59%) | 0.068 |
| Weight, kg | 76 (65–85) | 77 (68–84) | 0.175 |
| Body mass index, kg/m^2^ | 25.0 (22.5–28.0) | 26.1 (23.9–31.9) | 0.024 |
| **Comorbidities, n (%)** |  |  |  |
| Hypertension | 181 (64.0%) | 33 (80.5%) | 0.037 |
| Atrial fibrillation | 44 (15.5%) | 9 (22.0%) | 0.300 |
| Peripheral vasculopathy | 19 (6.7%) | 8 (19.5%) | 0.006 |
| Type 2 diabetes mellitus | 16 (5.7%) | 6 (14.6%) | 0.033 |
| Dyslipidemia | 73 (25.8%) | 17 (41.5%) | 0.036 |
| History of cardiac surgery | 4 (1.4%) | 2 (4.9%) | 0.124 |
| **Baseline clinical data** |  |  |  |
| Leucocytes, ×10^9^/L | 6.4 (5.2–7.3) | 6.7 (5.8–8.2) | 0.048 |
| Hemoglobin, g/dL | 14.1 (13.2–15.1) | 13.9 (13.0–14.9) | 0.348 |
| Platelets, ×10^9^/L | 204 (179–241) | 209 (183–271) | 0.260 |
| Albumin, g/dL | 4.0 (3.8–4.1) | 3.9 (3.7–4.0) | 0.011 |
| eGFR, mL/min/1.73 m^2^ ^a^ | 92.0 (83–99) | 82 (72–92) | 0.001 |
| Urea, mg/dL ^b^ | 35 (30–42) | 40 (32–44) | 0.016 |
| Troponin I, μg/L | 0.01 (0.01–0.01) | 0.01 (0.01–0.01) | 0.850 |
| NYHA classification, n (%) |  |  | 0.053 |
| 1 | 110 (38.9%) | 8 (19.5%) |  |
| 2 | 166 (58.7%) | 32 (78.0%) |  |
| 3 | 7 (2.5%) | 1 (2.4%) |  |
| Left ventricular ejection fraction, % | 62.0 (58.0–67.0) | 61.0 (55.5–69.4) | 0.802 |
| Systolic pulmonary arterial pressure, mm Hg | 30.0 (26.0−37.0) | 30.0 (28.0−39.5) | 0.378 |
| EuroSCORE II for operative risk, %^c^ | 1.07 (0.69−1.81) | 1.03 (1.02−3.81) | <0.001 |
| STS Risk Score, %^d^ |  |  |  |
| Risk of mortality | 0.60 (0.33–0.99) | 1.35 (1.01–1.73) | <0.001 |
| Risk of morbidity or mortality | 8.22 (6.37−10.57) | 12.35 (9.77−14.29) | <0.001 |
| Risk of renal failure | 1.24 (0.87−1.97) | 2.28 (1.54−2.99) | <0.001 |
| Thakar Score^e^ |  |  | 0.032 |
| 0.4 | 228 (80.6%) | 27 (65.9%) |  |
| 1.8 | 55 (19.4%) | 14 (34.1%) |  |
| **Operative data** |  |  |  |
| Aortic cross-clamp, min | 79.5 (59.0−97.0) | 82.0 (61.5−124.5) | 0.080 |
| Cardiopulmonary bypass time, min | 112.0 (91.0−139.0) | 136.0 (101.0−166.0) | 0.013 |
| Procedure, n (%) |  |  |  |
| Coronary artery bypass graft only | 10 (3.5%) | 2 (4.9%) | 0.453 |
| Valve only | 145 (51.2%) | 20 (48.9%) | 0.412 |
| Combined or other | 125 (44.2%) | 20 (48.8%) | 0.301 |
| Minimally invasive, n (%) | 199 (70.3%) | 14 (34.1%) | <0.001 |
| Intraoperative diuresis, mL | 900 (550−1300) | 1100 (530−1460) | 0.653 |
| Surgery fluid balance, mL | 3650 (2860−4300) | 4250 (2945−4993) | 0.019 |
| Intraoperative contrast media, n (%) | 4 (1.4%) | 1 (2.4%) | 0.619 |
| Lowest mean arterial pressure, mm Hg | 66.7 (63.3−70.0) | 66.3 (61.8−70.0) | 0.404 |
| Lowest hemoglobin, g/dL | 9.9 (8.9−11.0) | 9.2 (7.6−10.5) | 0.014 |
| Red blood cell transfusion, n (%) | 13 (4.6%) | 4 (9.8%) | 0.102 |
| **ICU data** |  |  |  |
| Mechanical ventilation, days | 1.0 (1.0−1.0) | 1.1 (1.0−1.2) | <0.001 |
| Intra-aortic balloon pump, n (%) | 3 (1.1%) | 5 (12.2%) | <0.001 |
| Extra-corporeal membrane oxygenation, n (%) | 0 (0%) | 3 (7.3%) | <0.001 |
| Myocardial infarction, n (%) | 0 (0%) | 1 (0.9%) | 0.231 |
| Stroke, n (%) | 0 (0%) | 4 (9.0%) | <0.001 |
| Re-intervention, n (%) | 3 (1.1%) | 5 (12.2%) | <0.001 |
| Cumulative fluid balance, mL | −1566 (−2597 to −586) | −1149 (−3314 to −27) | 0.342 |
| Weight difference, kg^f^ | −1.80 (−3.30 to −0.20) | -0.00 (−1.80 to 2.05) | <0.001 |
| ACEi or ARB use, n (%) | 65 (23.0%) | 10 (24.4%) | 0.840 |
| Aminoglycoside use, n (%) | 2 (0.7%) | 0 (0%) | 0.589 |
| Vancomycin use, n (%) | 2 (0.7%) | 1 (2.4%) | 0.279 |
| NSAID drug use, n (%) | 3 (1.1%) | 1 (2.4%) | 0.455 |
| Mean arterial pressure <65 mm Hg  within the first 24 hours, n (%) | 93 (32.9%) | 25 (61.0%) | <0.001 |
| Inotropes, n (%) | 88 (31.1%) | 20 (48.8%) | 0.025 |
| ICU stay, h | 43 (41−71) | 88.0 (62−162) | <0.001 |
| Hospital stay , days | 6 (5−7) | 7 (6−10) | 0.004 |
| **3-month follow-up data** |  |  |  |
| Readmission, n (%) | 5 (1.4%) | 4 (9.8%) | 0.004 |
| 3-month mortality, n (%) | 0 (0%) | 1 (2.4%) | 0.009 |
| **1-year follow-up data** |  |  |  |
| 1-year mortality, n (%) | 0 (0%) | 3 (7.3%) | <0.001 |

^†^ Summaries of quantitative variables are presented as median and inter-quartile range (in parentheses). For categorical variables, the absolute and relative frequencies (as %, in parentheses) for the categories are presented.

^a^ eGFR was calculated with the Chronic Kidney Disease-Epidemiology Collaboration equation [2].

^b^ To convert the value for urea to blood urea nitrogen, multiply by 0.467.

^c^ The European System for Cardiac Operative Risk Evaluation (EuroSCORE) score is calculated by means of a logistic-regression equation and ranges from 0 to 100%, with higher scores indicating greater risk.

^d^ The STS Risk Score is calculated by means of a logistic-regression equation; it estimates the risk of morbidity and mortality and the risk of renal failure, and ranges from 0 to 100% (higher scores indicate greater risk).

^e^ The Thakar Score is calculated by means of a logistic-regression equation; it estimates the risk of dialysis for patients undergoing cardiac surgery, and ranges from 0 to 21.5% (higher scores indicate greater risk).

^f^ Difference of ICU discharge weight and hospital admission weight.

ACEi, angiotensin-converting enzyme inhibitor; AKI, acute kidney injury; ARB, angiotensin II receptor blocker; eGFR, estimated glomerular filtration rate; ICU, intensive care unit; IQR, interquartile range; NSAID, non-steroidal anti-inflammatory drug; NYHA, New York Heart Association; STS, Society of Thoracic Surgeons.

## Table S3: Serum creatinine levels among patients receiving high oral protein loading, as compared to those in patients receiving standard care prior to cardiac surgery

(a) Serum creatinine values (in mg/dL)^a^

|  | **Control group** | **Protein-loading group** | **Difference** (protein-loading vs. control) | **p-value (unadjusted)** | **p-value (adjusted)^b^** |
| --- | --- | --- | --- | --- | --- |
| **3 months** |  |  |  |  |  |
| All patients (n = 323) | 0.89 [0.86–0.91] | 0.85 [0.82–0.88] | −0.04 [−0.08–0.01] | 0.088 | 0.048 |
| No AKI (n = 283) | 0.89 [0.86–0.91] | 0.84 [0.80–0.87] | −0.05 [−0.09– −0.01] | 0.023 | 0.019 |
| AKI (n = 40) | 0.88 [0.79–0.96] | 0.93 [0.82–1.0] | 0.06 [−0.08–0.20] | 0.411 | 0.651 |
| Reversal^c^ (n  = 35) | 0.84 [0.75–0.93] | 0.93 [0.82–1.0] | 0.09 [−0.05–0.23] | 0.207 | 0.341 |
| Non-reversal | 1.00 [0.87–1.73] | - | - | - |  |
| **12 months** |  |  |  |  |  |
| All patients (n = 321) | 0.96 [0.94–0.98] | 0.87 [0.84–0.91] | −0.09 [−0.13– −0.05] | <0.001 | <0.001 |
| No AKI (n = 283) | 0.95 [0.92–0.97] | 0.86 [0.82–0.89] | −0.09 [−0.13– −0.05] | <0.001 | <0.001 |
| AKI (n = 38) | 1.10 [0.97–1.20] | 0.97 [0.83–1.10] | −0.10 [−0.28–0.07] | 0.243 | 0.184 |
| Reversal^c^ (n  = 34) | 0.99 [0.90–1.10] | 0.97 [0.86–1.10] | −0.02 [−0.16–0.12] | 0.788 | 0.487 |
| Non-reversal | 1.50 [1.00–2.00] | - | - | - |  |

(b) Change in serum creatinine levels (in mg/dL)^a^ relative to the preoperative values

|  | **Control group** | **Protein-loading group** | **Difference** (protein-loading vs. control) | **p-value (unadjusted)** | **p-value (adjusted)^b^** |
| --- | --- | --- | --- | --- | --- |
| **3 months** |  |  |  |  |  |
| All patients (n = 323) | 0.04 [0.03–0.05] | 0.01 [−0.01–0.03] | −0.03 [−0.06– −0.01] | 0.006 | 0.006 |
| No AKI (n = 283) | 0.04 [0.03–0.05] | 0.00 [−0.02–0.02] | −0.04 [−0.06– −0.01] | 0.002 | 0.002 |
| AKI (n = 40) | 0.04 [−0.01–0.08] | 0.04 [−0.02–0.10] | 0.00 [−0.07–0.08] | 0.903 | 0.979 |
| Reversal (n  = 35) | 0.00 [−0.04–0.04] | 0.04 [−0.01–0.09] | 0.04 [−0.03–0.11] | 0.235 | 0.375 |
| Non-reversal | 0.18 [0.03, 0.34] | - | - | - |  |
| **12 months** |  |  |  |  |  |
| All patients (n = 321) | 0.11 [0.10–0.13] | 0.03 [0.01–0.05] | −0.08 [−0.11– −0.06] | <0.001 | <0.001 |
| No AKI (n = 283) | 0.10 [0.08–0.11] | 0.02 [0.00–0.04] | −0.08 [−0.10– −0.05] | <0.001 | <0.001 |
| AKI | 0.23 [0.14–0.31] | 0.08 [−0.03–0.19] | −0.15 [−0.29– −0.00] | 0.045 | 0.060 |
| Reversal^b^ (n  = 34) | 0.14 [0.09–0.19] | 0.08 [0.02–0.14] | −0.06 [−0.14–0.01] | 0.101 | 0.100 |
| Non-reversal | 0.65 [0.09–1.20] | - | - | - |  |

Summaries of quantitative variables are presented as mean and 95% confidence interval (in brackets).

^a^ To convert the values for serum creatinine (in mg per deciliter) to micromoles per liter, multiply by 88.4.

^b^ P-values were taken from linear models, including age, sex, body mass index, hypertension, and diabetes as covariables.

^c^ Reversal of AKI was defined as the absence of any stage of AKI based on either the serum creatinine or urine output criteria at hospital discharge [6].

# AKI, acute kidney injury. SUPPLEMENTARY REFERENCES

1. Husain-Syed F, Ferrari F, Sharma A, Danesi TH, Bezerra P, Lopez-Giacoman S, Samoni S, de Cal M, Corradi V, Virzi GM, et al: Preoperative Renal Functional Reserve Predicts Risk of Acute Kidney Injury After Cardiac Operation. Ann Thorac Surg. 2018;105:1094-1101.

2. Levey AS, Stevens LA, Schmid CH, Zhang YL, Castro AF, 3rd, Feldman HI, Kusek JW, Eggers P, Van Lente F, Greene T, et al: A new equation to estimate glomerular filtration rate. Ann Intern Med. 2009;150:604-612.

3. Kidney Disease: Improving Global Outcomes (KDIGO) Acute Kidney Injury Work Group. KDIGO Clinical Practice Guideline for Acute Kidney Injury. Kidney Int Suppl. 2012:1-138.

4. Thielmann M, Kottenberg E, Kleinbongard P, Wendt D, Gedik N, Pasa S, Price V, Tsagakis K, Neuhauser M, Peters J, et al: Cardioprotective and prognostic effects of remote ischaemic preconditioning in patients undergoing coronary artery bypass surgery: a single-centre randomised, double-blind, controlled trial. Lancet. 2013;382:597-604.

5. Valle R, Aspromonte N, Milani L, Peacock FW, Maisel AS, Santini M, Ronco C: Optimizing fluid management in patients with acute decompensated heart failure (ADHF): the emerging role of combined measurement of body hydration status and brain natriuretic peptide (BNP) levels. Heart Fail Rev. 2011;16:519-529.

6. Kellum JA, Sileanu FE, Bihorac A, Hoste EA, Chawla LS: Recovery after Acute Kidney Injury. Am J Respir Crit Care Med. 2017;195:784-791.
